# Copper regulates the expression of immune genes in microglial cells *in vitro*

**DOI:** 10.1016/j.imbio.2025.153145

**Published:** 2025-12-02

**Authors:** Laura Craciun, Sandra E. Muroy, Kaoru Saijo

**Affiliations:** Department of Molecular and Cell Biology, University of California, Berkeley, Berkeley, CA, USA

**Keywords:** Microglia, Copper, Immunity, Gene expression analysis

## Abstract

Copper, a transition metal, plays crucial roles in various physiological functions, including those of the nervous and immune systems. Dysregulation of copper homeostasis is linked to several diseases, such as neurodegenerative diseases. Since dysfunctional microglial immunity can also contribute to such diseases, we investigated the role of copper in microglial immunity. Currently, the roles of copper in microglial immunity are considered complex and multifaceted, with both anti- and pro-inflammatory effects having been proposed. In the current study, we found that both increased and decreased copper levels suppressed lipopolysaccharide (LPS)-mediated inflammation in microglial cells, as determined by RT-qPCR analysis. RNA sequencing (RNA-seq) analysis confirmed that increased copper levels reduced the inflammatory response to LPS; however, it also showed that decreased copper levels affected genes involved in cell proliferation, transcription, and autophagosome regulation. These findings suggest that copper is vital for maintaining normal immune functions in microglia, and that both copper excess and deficiency can disrupt microglial immunity.

## 1. Introduction

Copper is the third most abundant metal in the human body (Bost et al., 2016; Festa and Thiele, 2011; Tapiero et al., 2003; Uauy et al., 1998). It regulates critical physiological functions in many systems, including the nervous and immune systems (Ackerman and Chang, 2018; Desai and Kaler, 2008; Gale and Aizenman, 2024; Locatelli and Farina, 2025; Murdoch and Skaar, 2022; Opazo et al., 2014; Stafford et al., 2013). Cells adapt to changes in extracellular copper concentration by rapidly regulating copper influx and efflux through specialized transporters and regulatory mechanisms (Curnock and Cullen, 2020; Guo et al., 2025; Yang et al., 2024). Such adaptation involves the coordinated action of copper importers, such as copper transporter 1 (CTR1), intracellular copper chaperones, and the exporters ATPase copper transporting alpha and beta (ATP7A and ATP7B, respectively), in addition to copper-responsive trafficking and sequestration systems.

CTR1, encoded by the solute carrier family 31 member 1 (*SLC31A1*) gene, is located on the plasma membrane and predominantly mediates copper intake (Lee et al., 2002; Nose et al., 2010). In contrast, ATP7A and ATP7B are the main copper exporters. They are located on the Golgi apparatus and sort copper into the lumen of the secretory compartment to be loaded onto cuproenzymes, such as ceruloplasmin (Robinson and Winge, 2010). ATP7A and ATP7B can also move to the plasma membrane for copper export when intracellular levels of copper are high (Telianidis et al., 2013). Although ATP7A and ATP7B have similar functions, they are not biologically redundant (Lutsenko et al., 2007; Schmidt et al., 2018), and their tissue-specific expression patterns differ. ATP7A is widely expressed in most organs, except the liver, while ATP7B is primarily expressed in the liver and, to a lesser extent, the kidney and brain (Chun et al., 2017; Terada et al., 1998; Yu et al., 2018). Mutations in these transporters result in rare genetic disorders, such as Menkes disease (caused by defective ATP7A), and Wilson’s disease (caused by alterations in ATP7B). Menkes disease is characterized by progressive neurodegeneration, connective tissue abnormalities, the presence of characteristic sparse and twisted hair, and other manifestations, such as hypotonia in infancy progressing to spasticity, feeding difficulties, seizures, failure to thrive, and skeletal abnormalities (Tumer and Moller, 2010; Zlatic et al., 2015). The symptoms of Wilson’s disease include hepatic dysfunction (elevated liver enzymes, hepatitis, cirrhosis, and liver failure), neurological abnormalities (movement disorders such as tremor, dystonia, parkinsonism, ataxia, chorea, and dysarthria), psychiatric manifestations (mood changes, irritability, depression, cognitive impairment), and ocular findings such as Kayser-Fleischer rings and cataracts (Czlonkowska et al., 2018; Shribman et al., 2019). Copper dysregulation is also linked to many neurodegenerative diseases, such as Alzheimer’s disease, Parkinson’s disease, and amyotrophic lateral sclerosis (ALS) (Desai and Kaler, 2008; Chen et al., 2022; Gorska et al., 2023; Gromadzka et al., 2020; Mezzaroba et al., 2019). Overall, this evidence suggests that copper is critical for maintaining normal physiological functions, particularly within the central nervous system (CNS).

Microglial cells are resident innate immune cells in the central nervous system (CNS) that protect the CNS from infection and injury. They also play critical roles in brain development and maintaining CNS homeostasis (Kettenmann et al., 2011; Li and Barres, 2018; Prinz et al., 2019). Neuroinflammation associated with dysfunctional microglia-mediated immune responses is observed in many neurological diseases, including neurodegenerative diseases (Gao et al., 2023; Hickman et al., 2018; Muzio et al., 2021). Although the impact of copper dysregulation in microglia is not well understood, many reports suggest that copper plays critical roles in other innate immune cells, such as macrophages. Copper has been shown to act in a proinflammatory manner by increasing the production of cytokines, reactive oxygen species (ROS), and reactive nitrogen species (RNS), as well as recruiting phagocytic cells (Flemming, 2023; Gao and Zhang, 2023; Kitazawa et al., 2016; Vo et al., 2024). In addition, it has been shown to change the epigenetic status of immune cells and promote inflammation (Flemming, 2023; Solier et al., 2023). However, other studies have suggested that copper suppresses inflammation and improves symptoms associated with chronic inflammatory diseases (Berthon, 1993; Zangiabadi et al., 2023). Therefore, exactly how copper regulates innate and microglial immunity is still unclear.

To understand how copper regulates LPS-mediated immune responses *in vitro*, we performed RT-qPCR analyses to examine the expressions of key proinflammatory mediators, including interleukin 1 beta (*Il-1β*), interleukin 6 (*Il-6*), and nitric oxide synthase 2 (*Nos2*), which are known to be upregulated in neuroinflammatory diseases (Chen et al., 2016; Zhang et al., 2023). In this initial screening, we found that both elevated and depleted copper levels suppressed the transcription of these proinflammatory mediators in a mouse microglial cell line following short-term lipopolysaccharide (LPS) stimulation. To further characterize the global effects of copper on microglial immunity, we conducted bulk RNA sequencing (RNA-seq). Consistent with the RT-qPCR results, our RNA-seq analysis revealed that excessive copper reduced the transcription of proinflammatory genes, including *Il-1β* and interferon beta (*Ifnβ*)-responsive genes, in response to LPS stimulation. In contrast, copper depletion attenuated the expression of genes associated with cell proliferation (*e.g*., *Cdk2ap2*, *Rb1*, and *Rbl2*) and autophagy (*e.g*., *Atg4a*, *Atg8*, *Atg13*, and *Vcp*). Together, these findings suggest that copper plays a critical role in regulating immune functions in microglial cells. Furthermore, a deeper mechanistic understanding of copper-mediated regulation of microglial immunity may provide important insights into the development and progression of copper dysregulation-associated neuroinflammatory diseases.

## 2. Materials and Methods

### 2.1. Cell Culture

The SIM-A9 mouse microglial cell line was kindly provided by Dr. Nagamoto-Combs (Nagamoto-Combs et al., 2014). The cells were maintained in DMEM/F12 medium (Corning) supplemented with 10 % fetal bovine serum (FBS; Hyclone), 5 % horse serum (Hyclone), and 1 % penicillin-streptomycin (Thermo Fisher Scientific).

### 2.2. Cell treatments

Lipopolysaccharide (LPS; 0.1 μg/mL; Sigma-Aldrich), (*N*(4)-methylthiosemicarbazonato)-Cu^II^ (Cu^II^(atsm)) (1 μM; Sigma-Aldrich), and tetrathiomolybdate (TTM) (20 μM, Sigma-Aldrich) were used to treat SIM-A9 cells, as indicated in the figure legends. For controls, we used the solvents for the drugs: DMSO (Sigma-Aldrich) for Cu^II^(atsm) and Dulbecco’s phosphate buffered saline (DPBS) (Thermo Fisher) for TTM. The average copper concentration in the human body is approximately 1.4–2.1 mg/kg body weight, corresponding to about 50–150 mg in an average adult. Approximately 9 % of total body copper is distributed in the brain (5.2 μg/g brain tissue) (Focarelli et al., 2022). In our tissue culture system, the DMEM/F12 medium contains about 330 ng/mL copper. In addition, the serum supplements used for SIM-A9 cell culture also contribute to the copper content. Specifically, we used DMEM/F12 supplemented with 10 % fetal bovine serum (FBS) and 5 % horse serum, which together provide an estimated additional 5–10 ng/mL copper. Considering that human serum contains approximately 1–1.5 μg/mL copper, the total copper concentration in our culture medium fulfills the essential requirement for cellular growth while remaining well below toxic levels (>5 μg/mL). To confirm that both TTM and Cu^II^(atsm) modulated intracellular copper concentrations in our system, we initially intended to use CD649 dye (Lee et al., 2020) to measure changes in intracellular copper concentration; however, this reagent is no longer available. As an alternative, we examined intracellular copper responses through bulk RNA-seq analysis of *Hmox1* (heme oxygenase-1) and *Hspa1a* (heat shock 70 kDa protein 1A) mRNA expression (Supplemental Fig. 1A and B), genes that regulate copper-mediated stress (Hufnagel et al., 2020; Muller et al., 2007). Our results showed that the expressions of these genes were altered as expected.

### 2.3. RNA-extraction and RT-qPCR

Previous studies have indicated that various factors can affect cellular responses to LPS stimulation—including intrinsic cellular heterogeneity (Barbour et al., 1998; Buscher et al., 2017; Naigles et al., 2023), technical variability (Kemfack et al., 2022; Wilfinger et al., 2023), circadian rhythms (Keller et al., 2009; Wang et al., 2016; Zeng et al., 2024), cell cycle stages (D’Angelo et al., 2017), and microenvironmental factors such as cell density and passage number (Cohly et al., 2001; Taciak et al., 2018). Therefore, 3 independent RT-qPCR experiments were performed on SIM-A9 cells to determine LPS-mediated responses in microglia. These experiments were completed on different days using cells at different passages to ensure reproducibility. Specifically, two experiments were performed on cells originating from the same vial (one experiment at passage 2–4 and one experiment at passage 6–8) and one experiment was performed on cells from a new vial (at passage 2–4). For each experiment, we plated two wells (24-well plate) of SIM-A9 cells per condition to assess consistency; thus, *n* = 6 wells/condition across the three experiments. RNA was purified and cDNA was generated for each well. Each cDNA sample was then used for RT-qPCR analysis by preparing two qPCR reactions per sample (technical duplicates). Technical duplicates were averaged to get an mRNA expression value for each well. Cycle threshold (Ct) values of the target genes were normalized to the housekeeping gene hypoxanthine phosphoribosyltransferase (*Hprt*), and fold changes were calculated using the ΔΔCt method. We chose Hprt as the reference gene based on our validation experiments showing that its expression did not significantly change upon LPS stimulation and because published studies reported similar observations (Bian et al., 2017; Boda et al., 2009; Fazzina et al., 2024; Selvey et al., 2001). For our analyses, we determined the log_2_ fold change in mRNA expression for each well compared to the control treatment and 0 h LPS stimulation and then graphed the results as the mean log_2_FC ± standard deviation (SD) of all 6 wells.

Total RNA was isolated using the Quick-RNA Miniprep Kit (Zymo Research). Complementary DNA (cDNA) was synthesized using the SuperScript III Kit (Thermo Fisher). Quantitative PCR (qPCR) was performed using SYBR Green Master Mix (Roche) on the QuantStudio 6 Real-Time PCR System (Thermo Fisher). Primers for the RT-qPCR assays were obtained from PrimerBank-MGH-PGA, and their sequences are summarized in Supplemental Table 4.

### 2.4. Generation of shRNA cell lines

The tet-on inducible lentivirus vector pTRIPZ (Dharmacon) was used to generate the *Slc31a1* (encodes CTR1) and *Atp7a* knockdown cell lines. We used two different shRNA sequences each to knock down *Slc31a1* and *Atp7a*, and we used a luciferase-specific sequence as a non-targeting control. Sequences for the shRNAs are listed in Supplemental Table 5. Lentiviruses were generated by co-transfecting each pTRIPZ targeting vector along with pCMV-VSV-G (Addgene plasmid #8454) and pCMV-dR8.2 (Addgene plasmid #8455) packaging plasmids into HEK 293 T cells using Lipofectamine 3000 reagent (Life Technologies), according to the manufacturer’s instructions. Supernatant containing virus was harvested after 48 h and added to SIM-A9 cells. Forty-eight hours post-transduction, puromycin (8 μg/ml) was added to select for transduced cells. Successful transduction was checked after 24–48 h of treatment with 0.5 μg/ml doxycycline by (1) detecting the presence of RFP+ cells *via* FACS on an Aria Fusion (BD Biosciences, UC Berkeley Cancer Research Laboratory) and (2) determining the knockdown efficiency of the shRNAs *via* RT-qPCR. Following the confirmation of transduction/gene knockdown, each cell line was expanded and subcultured for immune stimulation experiments, as described.

### 2.5. Bulk RNA-sequencing

SIM-A9 cells were pretreated with compound for 1 h followed by LPS for 12 h before RNAs were extracted, as described above. RNA-seq libraries were prepared and sequenced using the NovaSeq PE 150 system by Novogene.

### 2.6. Bioinformatic analysis

Bioinformatic analysis of the RNA-seq data was performed as described by Goto et al. (2022). Briefly, reads were trimmed with Cutadapt 3.4 to remove the 3′ adapter sequences. Quality control was then performed using FastQC 0.11.7. Reads were aligned to the GRCm38-mm10 *mus musculus* reference genome using Spliced Transcripts alignment to Reference aligner 2.7.1a. TPMCalculator 0.0.4 was used to generate count data, and we performed gene expression analysis with DEseq2 1.32.0. Genes with a false discovery rate (FDR) < 0.1 were considered differentially expressed. Gene set enrichment analysis (GSEA) was performed on the sets of differentially expressed genes to identify enriched biological themes. Volcano plots were generated, and the GO analyses were performed using RStudio (R 3.6.0+).

### 2.7. Statistical analysis

The RT-qPCR data for LPS-mediated responses are presented as means (log_2_FC) ± standard deviation (SD). Other RT-qPCR data are shown as relative mRNA expression normalized to the control treatment. Statistical significance was assessed using one-way or two-way ANOVA to evaluate the effects of treatment and/or time, as well as their interaction. Interaction F (DFn, DFd) values and corresponding *P*-values are provided in the figure legends, and all ANOVA results are presented in the Supplemental Information. When a significant interaction was detected, simple effects were examined using Tukey’s multiple comparisons post-hoc test. All statistical analyses were performed using GraphPad Prism (version 10). A *P-value* < 0.05 was considered statistically significant.

## 3. Results

### 3.1. Decreasing copper levels reduces LPS-mediated inflammation

**Fig. 1.**
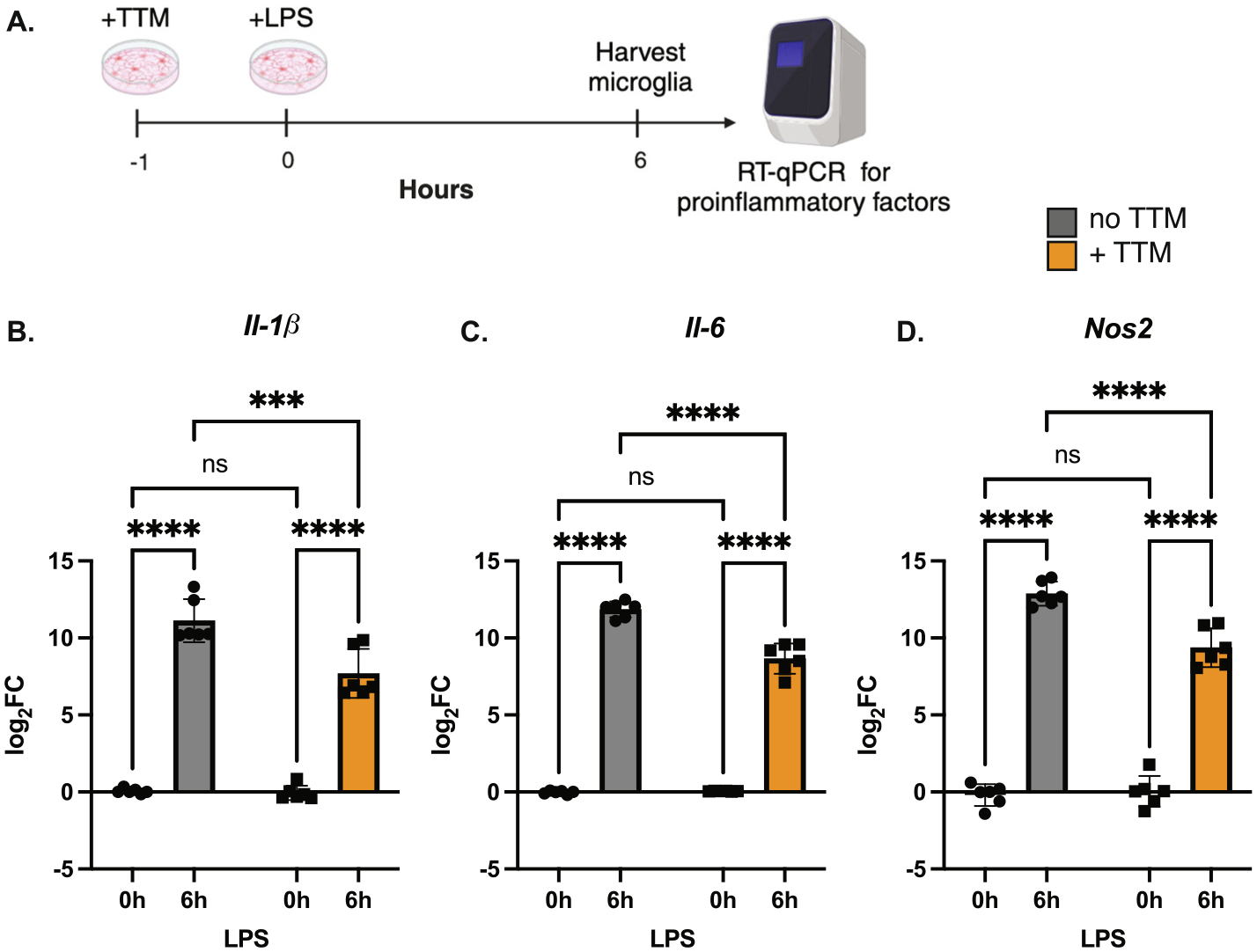
TTM-treated SIM-A9 cells (decreased copper) show reduced transcription of proinflammatory factors. A) Experimental scheme is shown. The mRNA expressions of B) *Il-1β* (F (1,20) = 13.95, *P* = 0.0013), C) *Il-6 (F (1, 20)* = *51.79, P* < 0.0001), and D) *Nos2* (F (1, 20) = 22.66, *P* = 0.0001) were determined by RT-qPCR. Gray bars indicate control treatment (DPBS) (*n* = 6/condition), and orange bars indicate TTM treatment (*n* = 6/condition). Results are shown as mean log_2_ fold change (log_2_FC) ± standard deviation (SD). Data were analyzed using two-way ANOVA with Tukey’s multiple comparison post-hoc test. ***p < 0.001, **** *p* < 0.0001, and ns: not significant.

To investigate the impact of copper levels on microglial immunity, we conducted *in vitro* experiments using SIM-A9 cells, a mouse microglial cell line. We first treated the cells with the copper chelator tetrathiomolybdate (TTM) (Kirk et al., 2024) for 1 h to reduce intracellular copper levels, followed by stimulation with lipopolysaccharide (LPS), a toll-like receptor 4 (TLR4) ligand (Ciesielska et al., 2021; Park and Lee, 2013), for 6 h (Fig. 1A). We selected *Il-1β*, *Il-6*, and *Nos2* for our initial screening because dysregulation of these genes has been well documented in previous studies (Chen et al., 2016; Zhang et al., 2023). As shown in Fig. 1B–D, the expressions of all three of these proinflammatory genes were decreased upon TTM treatment. This repression was not due to TTM toxicity, as the expression of the housekeeping gene *Hprt* remained unchanged (Supplemental Fig. 2A). In addition, since copper overload and, to a lesser extent, copper depletion are known to induce cell death (Chan et al., 2008), we also examined the expressions of *Bax* (Supplemental Fig. 2C) and *Bcl-xL* (Supplemental Fig. 2E), which are key regulators of apoptosis. We found that the expression levels of these proteins did not differ in TTM-treated cells compared to control cells. Thus, reducing intracellular copper levels prevents the normal induction of proinflammatory mediators upon LPS stimulation.

**Fig. 2.**
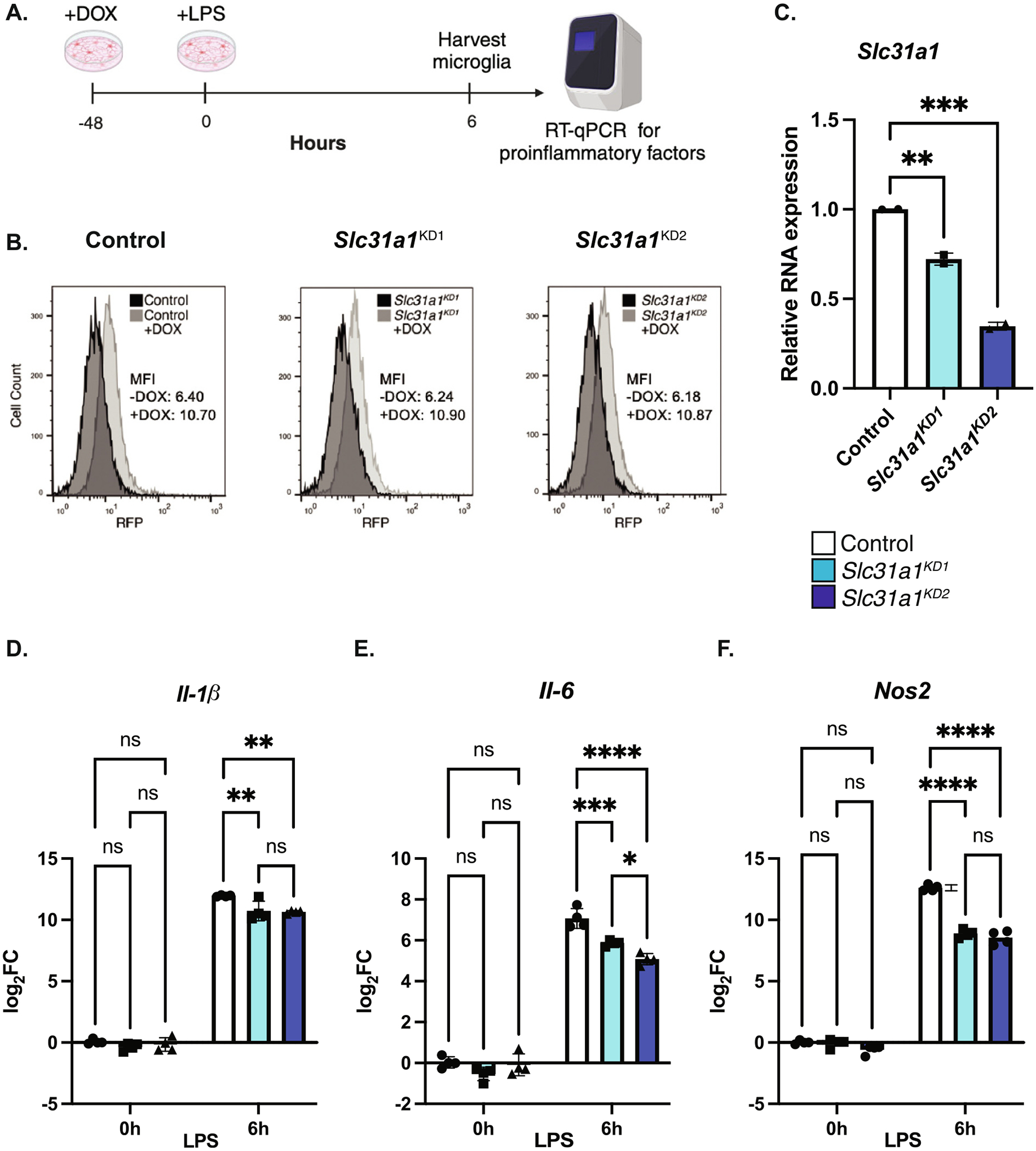
*Slc31a1* knockdown SIM-A9 cells show decreased expression of proinflammatory genes. A) Experimental scheme is shown. B) Doxycycline (DOX)-induced RFP expression in the transduced cell lines. Numbers indicate median fluorescence intensity (MFI). C) *Slc31a1* mRNA levels in control (white bar, *n* = 4), *Slc31a1*^KD1^ (light blue bar, *n* = 4), and *Slc31a1*^KD2^ (dark blue bar, *n* = 4) SIM-A9 cells are shown to indicate shRNA knockdown efficiency. F (2,3) = 398.3, *P* = 0.0002. The mRNA expressions of D) *Il-1β* (F (2, 18) = 3.551, *P* = 0.0501) E) *Il-6* (F (2, 18) = 13.50, *P* = 0.0003), and F) *Nos2* (F (2, 18) = 66.42, *P* < 0.0001) in SIM-A9 cell lines in response to LPS treatment were determined. Control (white bar, *n* = 4), *Slc31a1*^KD1^ (light blue bar, *n* = 4), and *Slc31a1*^KD2^ (dark blue bar, *n* = 4). Results are shown as mean log_2_ fold change (log_2_FC) ± standard deviation (SD) (D=F) or as means ± standard deviation (SD) (C). Data were analyzed using two-way ANOVA (D–F) or one-way ANOVA (C) with Tukey’s multiple comparison post-hoc test. *p < 0.05, **p < 0.01, ***p < 0.001, **** *p* < 0.0001, and ns: not significant.

Because the duration of the effects of TTM *in vitro* is unknown, we next sought to establish stable SIM-A9 cell lines with reduced intracellular copper levels by knocking down *Slc31a1*, which encodes the copper importer CTR1 (Lee et al., 2002; Nose et al., 2010). We used two different doxycycline-inducible short-hairpin RNAs (shRNAs) to generate two knockdown cell lines—*Slc31a1* knockdown 1 (*Slc31a1*^KD1^) and *Slc31a1* knockdown 2 (*Slc31a1*^KD2^)—along with a non-targeting shRNA control. For our experiments, cells were treated with doxycycline (Dox) for 48 h to induce *Slc31a1* knockdown, followed by 6 h of LPS stimulation to test the induction of proinflammatory mediators (Fig. 2A). As shown in Fig. 2B–C, successful transduction of the shRNAs was confirmed by RFP fluorescence, and both knockdown cell lines exhibited significantly reduced *Slc31a1* mRNA levels, reaching less than 50 % of what was observed in control cells. Consistent with the TTM results, *Slc31a1* knockdown led to significant decreases in the expressions of *Il-1β* (Fig. 2D), *Il-6* (Fig. 2E), and *Nos2* (Fig. 2F) in response to LPS. These data collectively indicate that reducing intracellular copper concentrations in microglia suppresses the induction of proinflammatory mediators upon LPS stimulation.

### 3.2. Increasing copper levels also reduces LPS-mediated inflammation

**Fig. 3.**
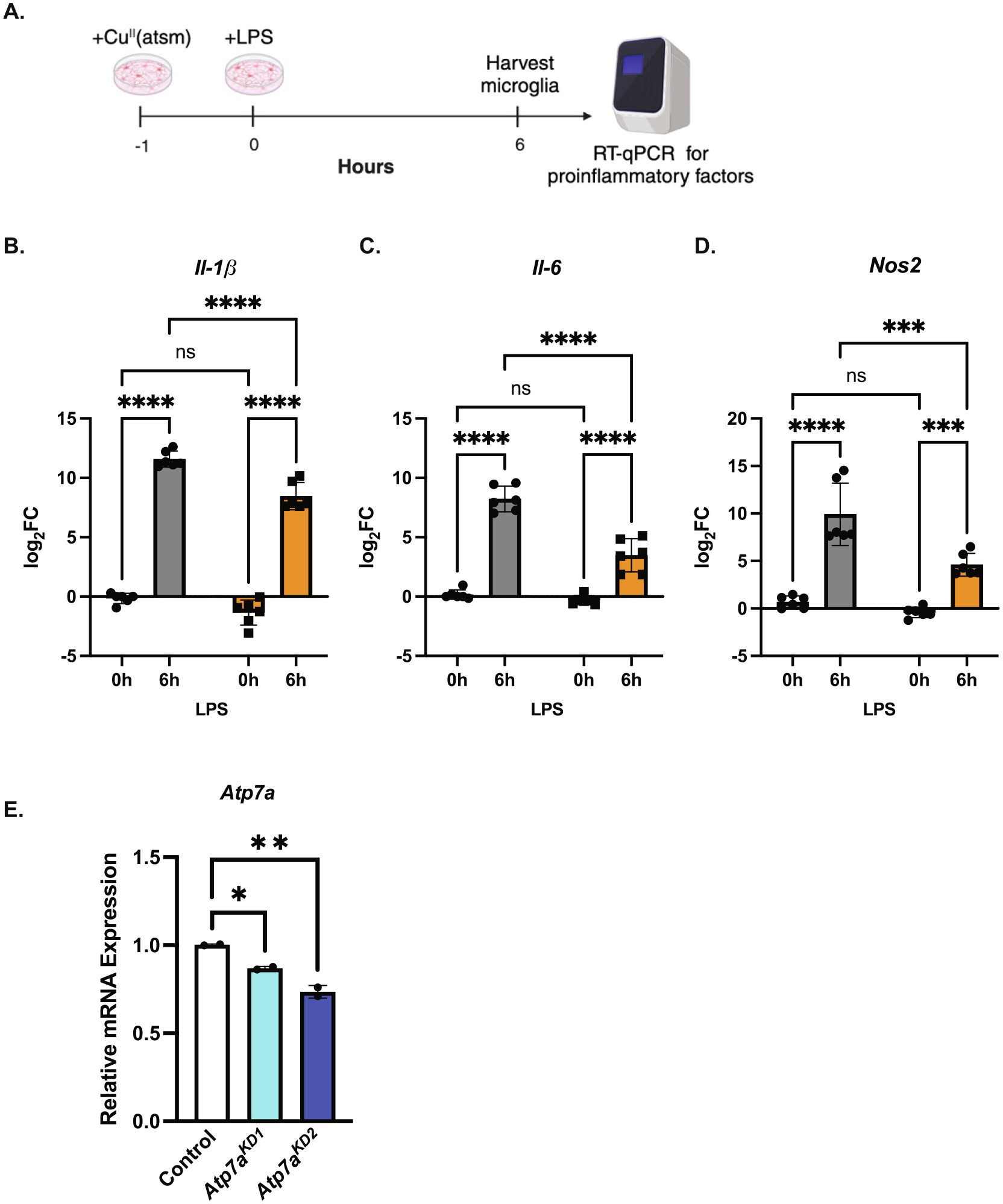
Cu^II^(atsm)-treated SIM-A9 cells (increased copper) show repressed proinflammatory gene expression. A) Experimental scheme is shown. The mRNA expressions of B) *Il-1β* (F (1, 20) = 7.187, *P* = 0.0144), C) *Il-6 (F (1, 20)* = *31.99, P* < 0.0001), and D) *Nos2* (F (1, 20), *P* = 0.0098) were determined by RT-qPCR. Gray bars indicate control treatment (DMSO, *n* = 6/condition), and orange bars indicate Cu^II^(atsm) treatment (*n* = 6/condition). E) The expression levels of *Atp7a* in control cells (white bar, *n* = 2), as well as the *Atp7a*^KD1^ (light blue bar, *n* = 2) and *Atp7a*^KD2^ (dark blue bar, *n* = 2) SIM-A9 shRNA knockdown cell lines are shown (F (2,3) = 75.46, *P* = 0.0027). Results are shown as mean log_2_ fold change (log_2_FC) ± standard deviation (SD) (B–D) or as means ± standard deviation (SD) (E). Data were analyzed using two-way ANOVA (B–C) or one-way ANOVA (E) with Tukey’s multiple comparison post-hoc test. *p < 0.05, **p < 0.01, ***p < 0.001, **** *p* < 0.0001, and ns: not significant.

Our previous work showed that microglial cells *in vitro* accumulate intracellular copper over time upon treatment with LPS (Lee et al., 2020). Therefore, we next tested how increasing intracellular copper levels affects LPS-mediated inflammatory gene expression in microglia. For these experiments, SIM-A9 cells were first pre-treated with diacetyl bis (*N*(4)-methylthiosemicarbazonato)-Cu^II^ (Cu^II^(atsm)) (Southon et al., 2020) for 1 h to increase the copper concentration inside the cells. This treatment was followed by 6 h of LPS stimulation and then RT-qPCR analysis to quantify proinflammatory gene expression (Fig. 3A). We found that the LPS-induced expressions of *Il-1β* (Fig. 3B), *Il-6* (Fig. 3C), and *Nos2* (Fig. 3D) were significantly lower when the cells were pre-treated with Cu^II^(atsm). We also observed that the induction of *Il-1α* expression was reduced by Cu^II^(atsm) (Supplemental Fig. 3A). Interestingly, the expression of tumor necrosis factor alpha (*Tnfα*) was not altered (Supplemental Fig. 3B), suggesting that in our experimental setting, Cu^II^(atsm) prevents the LPS-mediated induction of some proinflammatory mediators but not all. Similar to TTM, we did not note any cell toxicity or apoptosis with Cu^II^(atsm) treatment, as evidenced by no change in *Hprt* (Supplemental Fig. 2B), *Bax* (Supplemental Fig. 2D), or *Bcl-xL* (Supplemental Fig. 2F) expressions.

In addition to our compound studies, we also attempted to establish SIM-A9 cell lines wherein *Atp7a*, which encodes a transporter that exports copper from cells (Telianidis et al., 2013; Kaler, 2011), was knocked down in order to increase intracellular copper concentrations. For these experiments, we used two different doxycycline-inducible shRNAs targeting *Atp7a* to generate two knockdown cell lines, *Atp7a* knockdown 1 (*Atp7a*^KD1^) and *Atp7a* knockdown 2 (*Atp7a*^KD2^). We also created a control cell line harboring a non-targeting shRNA. Unfortunately, we were unable to establish cell lines with sufficient knockdown of *Atp7a*, as both experimental cell lines exhibited less than 50 % knockdown compared to control cells (Fig. 3E). Therefore, we could not replicate the results of our Cu^II^(atsm) experiments using this methodology.

### 3.3. RNA-sequencing analysis of the copper-mediated modulation of LPS responses

**Fig. 4.**
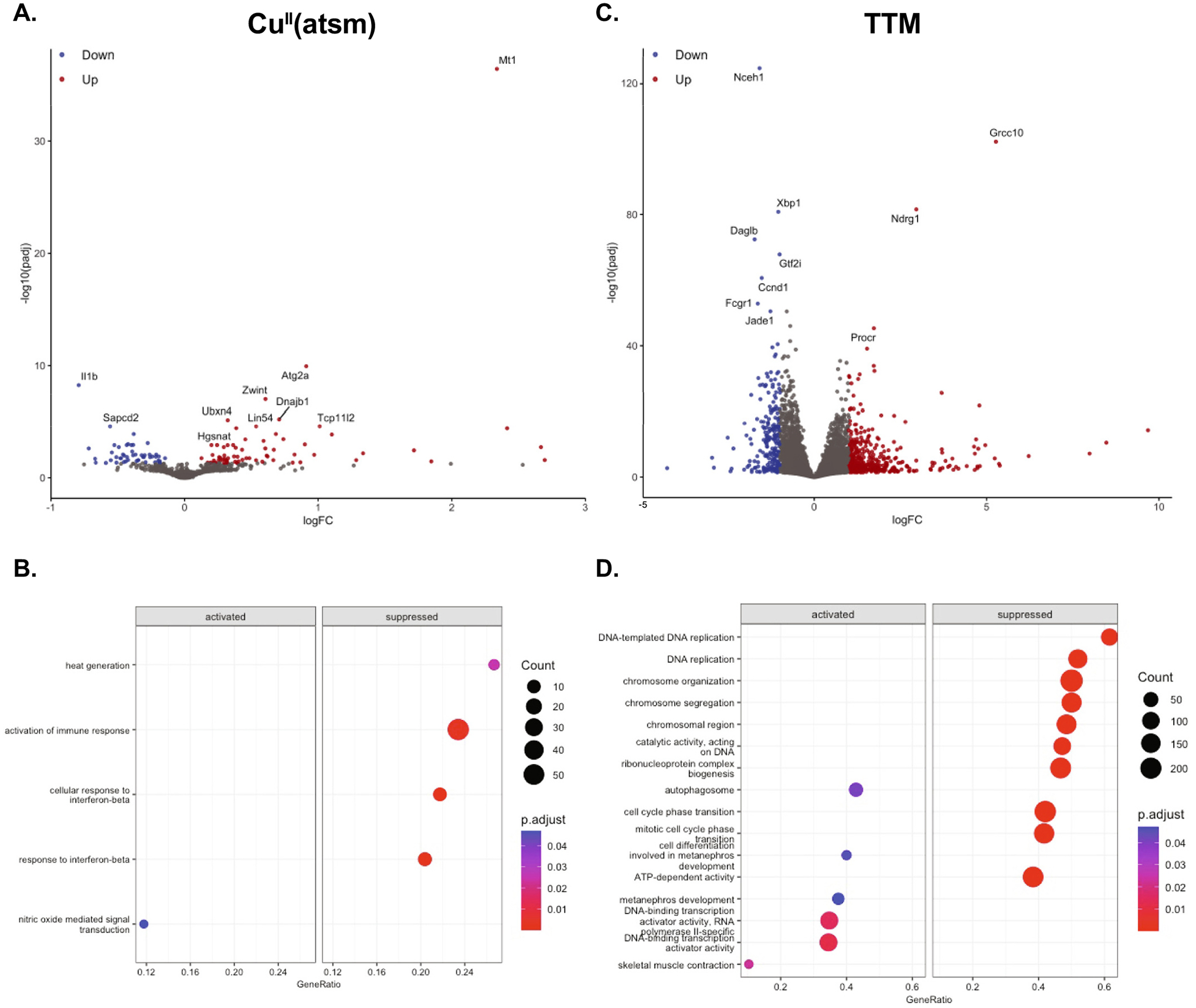
Increased and decreased copper concentrations regulate distinct subsets of genes upon LPS stimulation. A) Volcano plot indicating the differentially expressed genes (DEGs) identified in samples treated with 1 μM Cu^II^(atsm) and LPS for 12 h compared to samples treated with vehicle control (DMSO) and LPS for 12 h. The right side of the plot indicates increased expression, and the left side indicates decreased expression. Red and blue circles indicate genes with adjusted *p*-values (*p-adj*) less than or equal to 0.05 and log_2_ fold change (FC) values either greater than 1 or less than −1. The gray circles are genes that are not significant. B) GO analysis of the DEGs identified in A is shown. C) The volcano plot shown indicates DEGs identified in samples treated with 20 μM TTM and LPS for 12 h compared to vehicle control (DPBS) samples treated with LPS for 12 h. The right side of the plot indicates increased expression, and the left side indicates decreased expression. Red and blue circles indicate *p-adj* less than or equal to 0.05 and log_2_FC values either greater than 1 or less than −1. The gray circles are genes that are not significant. D) GO analysis of the DEGs identified in C is shown.

Since we found that both increasing and decreasing the level of copper in cells suppresses the induction of proinflammatory mediators upon LPS stimulation, we decided to perform bulk RNA-sequencing (RNA-seq) to examine the global gene expression changes mediated by copper during microglial inflammation. Previous studies have shown that the expressions of most proinflammatory mRNAs are rapidly induced by LPS stimulation, peaking within 6 to 12 h (Barrios-Rodiles et al., 1999; Xaus et al., 2000), which is consistent with what we observed in the microglial cells used in our RT-qPCR experiments (Supplemental Fig. 4A–C). In contrast, the induction of anti-inflammatory mRNAs occurs later, starting at around 12 h after treatment (Gurung et al., 2015). Therefore, for our RNA-seq studies, to capture a broader range of mRNAs that are regulated by LPS, we treated SIM-A9 cells with Cu^II^(atsm) or TTM for 1 h, followed by 12 h of LPS stimulation. Analysis of the RNA-seq data resulted in the identification of 118 genes that were significantly upregulated and 73 genes that were significantly downregulated (*p* adjusted <0.05, log_2_ fold change greater than 1 or less than −1, respectively) when the cells were treated with Cu^II^(atsm) prior to LPS (Fig. 4A, Supplemental Table 1). Consistent with our RT-qPCR analysis, we found that Cu^II^(atsm) prevented the LPS-mediated induction of proinflammatory genes, including *Il-1β, Fcgr1, Il12rb1, Tnsf12, and Tnsf13*. Gene ontology (GO) analysis of the differentially expressed genes (DEGs) indicated that the list of downregulated genes was enriched in immune response and IFN*β*-responsive genes, such as *Ifit3, Oas1b, Ifi47, and Calm* (Fig. 4B, Supplemental Table 1). Analysis of the upregulated genes did not identify any significant GO terms; however, nitric oxide signaling was slightly activated (Supplemental Table 1).

When SIM-A9 cells were pretreated with TTM before LPS stimulation, our RNA-seq analysis indicated that 1005 genes were significantly upregulated, and 638 genes were significantly downregulated (*p* adjusted <0.05, log_2_ fold change greater than 1 or less than −1, respectively) (Fig. 4C, Supplemental Table 2). Unlike our RT-qPCR results, we did not detect any proinflammatory genes whose expressions were significantly repressed by TTM with 12 h of LPS treatment. Subsequent GO analysis showed that the genes that were upregulated by TTM pretreatment included ones that regulate transcription (*Zwint*), proliferation (*Cdk2ap2*, *Rb1*, and *Rbl2*), and autophagosomes (*Ulk1*, *Atg4a*, *Atg8*, *Atg13*, and *Vcp*) (Fig. 4D, Supplemental Table 2). Pre-treatment with TTM also significantly reduced the LPS-induced expressions of genes involved in epigenetic regulation (*Smarcd1, Suv39h1*), and energy generation (*Sirt1*) (Fig. 4D, Supplemental Table 2). Overall, our RNA-seq results demonstrate that increasing copper concentrations suppresses the induction of proinflammatory mediators, while decreasing copper concentrations activates autophagosomes and suppresses cell proliferation.

### 3.4. Copper regulates critical immune functions induced by LPS

**Fig. 5.**
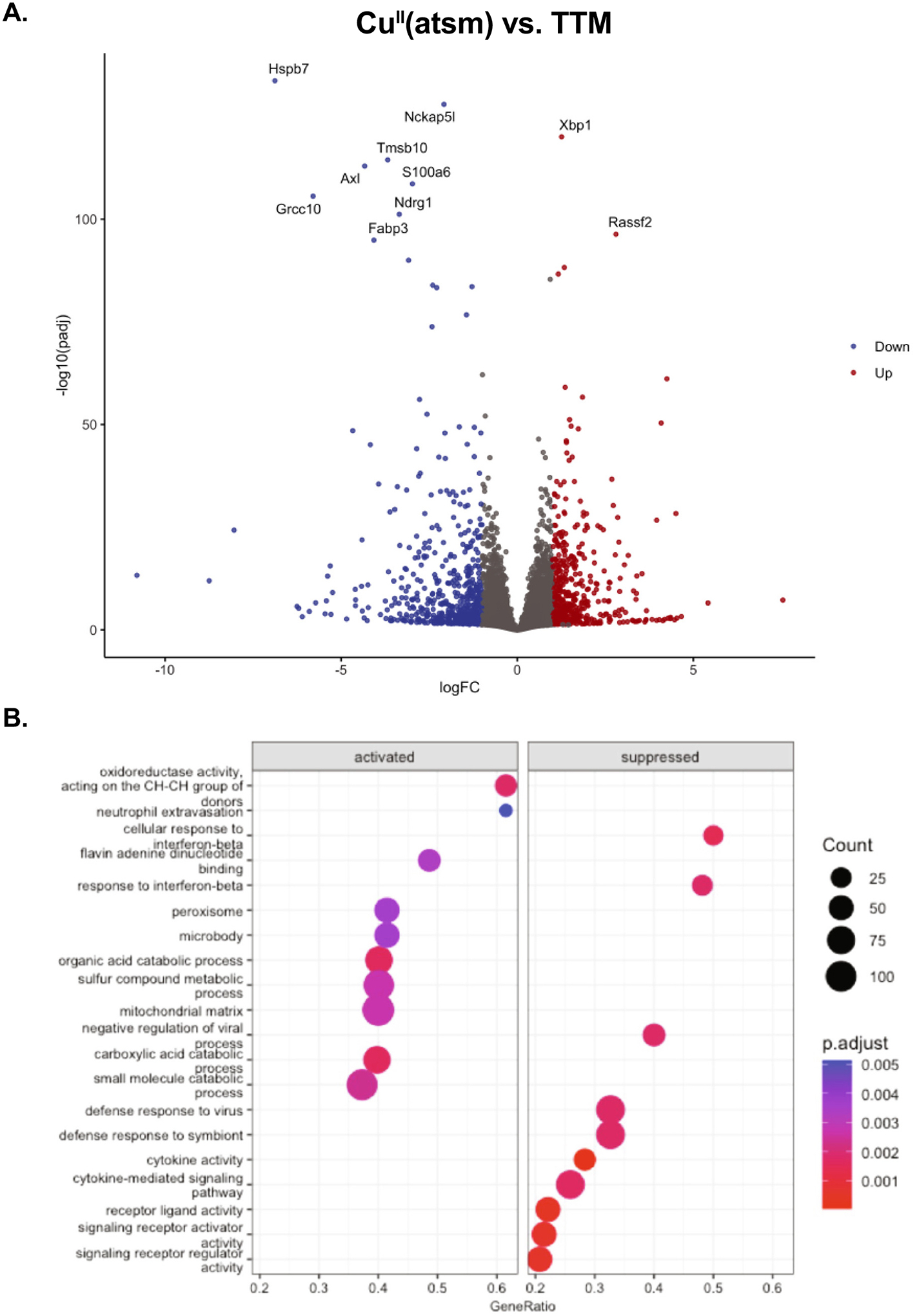
Copper regulates various immune functions in microglial cells. A) A volcano plot indicating the DEGs identified in samples treated with 1 μM Cu^II^(atsm) and 12 h of LPS compared to those treated with 20 μM TTM and 12 h of LPS is shown. The right side indicates DEGs whose expressions were increased by Cu^II^(atsm) compared to TTM, and the left side indicates DEGs whose expressions were decreased by Cu^II^(atsm) compared to TTM. Red and blue circles indicate *p-adj* less than or equal to 0.05 and log_2_FC values either greater than 1 or less than −1. The gray circles are genes that are not significant. B) GO analysis of the DEGs identified in A is shown.

To better understand the LPS-mediated immune functions impacted by increased and decreased copper concentrations, we directly compared gene expression in Cu^II^(atsm)-treated cells with gene expression in TTM-treated cells (*i.e*., the RNA-seq data from the TTM-treated cells was used as the reference for the differential gene expression analysis) (Supplemental Table 3). The left side of the volcano plot in Fig. 5A indicates the DEGs whose expressions were decreased by Cu^II^(atsm) relative to TTM, and the right side of the plot indicates the DEGs whose expressions were increased by Cu^II^(atsm) relative to TTM (*p* adjusted <0.05, log_2_ fold change greater than −1 or less than 1, respectively). GO analysis of these DEGs showed that Cu^II^(atsm) activated catabolism pathways and peroxisomes, compared to TTM (Fig. 5B). In addition, we found that Cu^II^(atsm) suppressed the expressions of IFN*β*-responsive genes as well as genes involved in cytokine signaling and phagocytosis, compared to TTM (Fig. 5B). These data suggest that both increased and decreased copper concentrations attenuate critical microglial immune functions *in vitro*.

## 4. Discussion

Copper dysregulation often contributes to the onset and progression of neurological disorders, including not only congenital diseases (Desai and Kaler, 2008; Chen et al., 2022; Kaler, 2011; La Fontaine and Mercer, 2007; de Bie et al., 2007) but also neurodegenerative diseases (Desai and Kaler, 2008; Chen et al., 2022; Gorska et al., 2023; Gromadzka et al., 2020; Mezzaroba et al., 2019). The role of copper as an anti- or proinflammatory agent in innate immune cells, such as macrophages, remains controversial (Gorska et al., 2023; Berthon, 1993; Hussain et al., 2019; Liu et al., 2022), and even less is known about copper regulation in microglia. Therefore, we investigated the roles of copper in microglial immune functions *in vitro*. Unexpectedly, we found that both increasing and decreasing intracellular copper concentrations in microglia using chemical compounds (Cu^II^(atsm) and TTM, respectively) led to significantly reduced expressions of proinflammatory genes in response to LPS. To confirm these results, we tried to generate cells with low and high levels of copper by knocking down the genes essential for copper import and export, respectively. Although we failed to establish a cell line with sufficient knockdown of *Atp7a* to reduce copper export (Fig. 3E), we did successfully generate cell lines with enough *Slc31a1* knockdown to reduce copper import. RT-qPCR analysis of the LPS response in these cells revealed proinflammatory gene expression patterns that were similar to those of parental cells treated with the copper chelator TTM (Figs. 1 and 2). Based on these findings, we realized a more global analysis of gene expression using RNA-seq was necessary to better understand the roles of copper in microglial immunity.

Consistent with our RT-qPCR results, our RNA-seq analysis showed that increasing intracellular copper levels with Cu^II^(atsm) reduced the expression of LPS-induced proinflammatory mediators, including cytokines and IFN*β*-responsive genes (Fig. 4A and B; Fig. 5). However, in contrast to our RT-qPCR analysis, our RNA-seq data showed that decreasing copper with TTM resulted in the suppression of genes that regulate cell proliferation and the activation of genes that regulate transcription and autophagosome formation, without any significant effects on inflammatory gene transcription (Fig. 4C and D; Fig. 5). One potential reason for the discrepancy between our RT-qPCR and bulk RNA-seq results is the difference in the timing of the LPS treatment. For many proinflammatory genes, 6 h of LPS treatment is good for detecting induction, but at 12 h, the induction of some genes might be saturated or may be decreasing due to resolution of the response. Conversely, some pro- and anti-inflammatory genes may require more time to activate or increase their expression (Supplemental Fig. 4), so 12 h of LPS treatment may improve the chance of detecting these genes, while 6 h of treatment may not be enough. Thus, a precise time course analysis of the gene expression changes in microglial cells in response to LPS may help us account for the differences we observed in our experiments.

Copper dysregulation has been reported in both Alzheimer’s and Parkinson’s disease (Chakraborty et al., 2015; Ladomersky and Petris, 2015; Shoudho et al., 2024). Copper levels and the expression levels of copper-associated proteins have also been shown to be altered in the spinal cords of patients with sporadic ALS (Kapaki et al., 1997; Min et al., 2024). Therefore, restoration of copper homeostasis has been proposed to help improve the symptoms of these diseases (Williams et al., 2016). Accordingly, Cu^II^(atsm) is currently in phase 2/3 clinical trials for the treatment of sporadic ALS (Hilton et al., 2024). However, exactly how copper in microglia contributes to these diseases has yet to be elucidated. Among the many functions of copper, redox regulation (generation of ROS and RNS (Chen et al., 2023, Yu et al., 2024)) is particularly interesting in terms of its potential for affecting physiopathology in humans. Aberrant generation of ROS and RNS is often associated with neurodegenerative disorders; thus, copper dyshomeostasis in microglia could greatly contribute to disease pathogenesis through the production of these highly reactive chemicals. Furthermore, neurodegenerative diseases are also associated with chronic neuroinflammation. Therefore, disruption of copper-mediated immune responses in microglia could play a key role in disease development and progression. Additional studies are needed to identify the specific copper pathways that contribute to neurodegenerative disease pathogenesis.

While copper has been shown to regulate microglia and other innate and adaptive immune cells (Liu et al., 2022; Cheng et al., 2022), other metals, such as iron and zinc, have also been shown to play critical roles in regulating immunity, and their dysregulation is also linked to neurodegenerative diseases (Ackerman and Chang, 2018; Ndayisaba et al., 2019; Shankar and Prasad, 1998; Szewczyk, 2013). Although all metals are essential for maintaining immune homeostasis and can act as antimicrobials (Shoudho et al., 2024; Mendes et al., 2022; Salah et al., 2021), some copper-specific features exist. For example, copper metabolism changes during inflammation, resulting in increased serum copper levels (Liu et al., 2022; Chakraborty et al., 2015). In addition, during infection, phagocytes increase copper uptake and mobilization, leading to high copper concentrations in phagolysosomes (Focarelli et al., 2022; Hodgkinson and Petris, 2012). Proinflammatory mediators also induce the expression of copper transporters, such as CTR1 and ATP7A, which facilitate copper uptake and delivery to the phagolysosome (Liu et al., 2022).

Although our results indicate that copper regulates various microglial immune functions, our study is limited by our *in vitro* tissue culture model. Several studies have suggested that the morphological and gene expression changes observed in microglia *in vitro* may not accurately reflect their function or state *in vivo*. Indeed, *in vitro* microglial cultures do not fully recapitulate the characteristics of adult human homeostatic microglia in that they appear more “activated” and have significantly different transcriptomic and morphological signatures compared to their *in vivo* counterparts (Maguire et al., 2022; Timmerman et al., 2018). Therefore, additional studies are needed to confirm the link between copper and microglial homeostasis *in vivo*. One compelling *in vivo* model involves stereotaxic injection of LPS into the substantia nigra—a procedure used by our group and others (Dutta et al., 2008; Saijo et al., 2009) to mimic neuroinflammation and Parkinson’s disease (PD) pathology. This model is particularly relevant given the well-documented copper dyshomeostasis observed in PD (Bisaglia and Bubacco, 2020; Montes et al., 2014). Examining gene expression changes in microglia in this model and assessing how copper pathway-targeting compounds alter microglial phenotypes could yield important insights into copper’s role in PD development and progression as well as the viability of these compounds as PD therapeutics. Future studies investigating whether modulating copper levels in microglia can produce beneficial effects in other *in vivo* neuroinflammatory disease models would also be valuable.

Despite its limitations, our *in vitro* model may still be useful for elucidating the pathological mechanisms underlying dysregulated copper homeostasis in disease. Earlier epidemiological studies have reported that copper exposure or increased copper intake might be associated with Parkinson’s disease (Zhao et al., 2023), suggesting that copper overload could contribute to PD onset. However, more recent studies have suggested that copper levels are actually decreased in this region (Montes et al., 2014; Genoud et al., 2017). If this is the case, supplementing with copper (*e.g*., Cu^II^(atsm)) may reduce inflammation and help improve symptoms. Therefore, a detailed time-course analysis, as well as co-culture experiments using microglia and neurons (*e.g*., dopaminergic neurons), could provide further insights into how copper temporally regulates microglial immunity and influences neuronal function in neurodegenerative diseases. Such information could be used to identify novel copper-related therapeutic targets in microglia or existing drugs that modulate copper-regulated pathways that have potential for minimizing neuroinflammation and preventing neurodegenerative disease progression.

## 5. Conclusion

We have found that increasing copper levels in microglia suppresses the expression of proinflammatory genes upon LPS stimulation, while decreasing copper levels alters the expression of genes involved in proliferation, transcription, and autophagosome regulation. Since our results indicate that precise regulation of intracellular copper levels is essential for normal microglial immune responses, targeting copper levels in microglia may be a potential therapeutic strategy for diseases associated with dysfunctional microglial immunity.

## Supplementary Material

1

2

3

4

5

6

7

8

9

10

## Data Availability

Data will be made available on request.

## Appendix A. Supplementary data

Supplementary data to this article can be found online at https://doi.org/10.1016/j.imbio.2025.153145.
