# Supplementary figures and images for: Copper regulates the expression of immune genes in microglial cells in vitro
Source: Immunobiology. Author manuscript; Available in PMC 2026 Jun 22. (PMC13285316; doi:10.1016/j.imbio.2025.153145)

Supplemental Fig. 1

A.

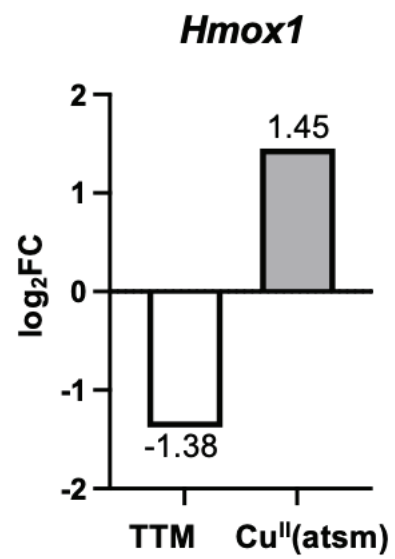

B.

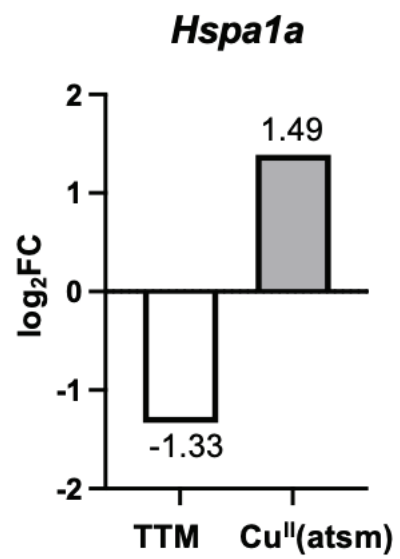

Supplement: 1 [file NIHMS2187441-supplement-1.pdf]

### Supplemental Fig. 2

**A.**

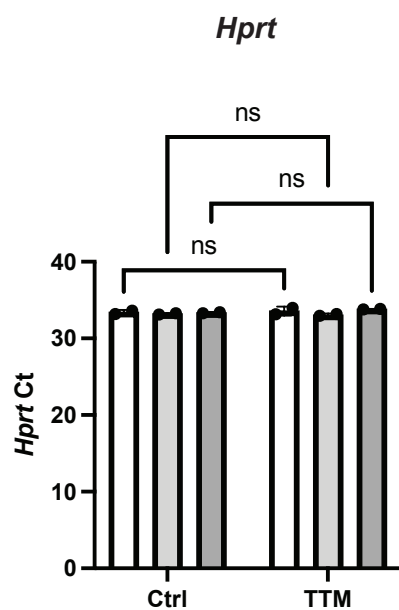

**B.**

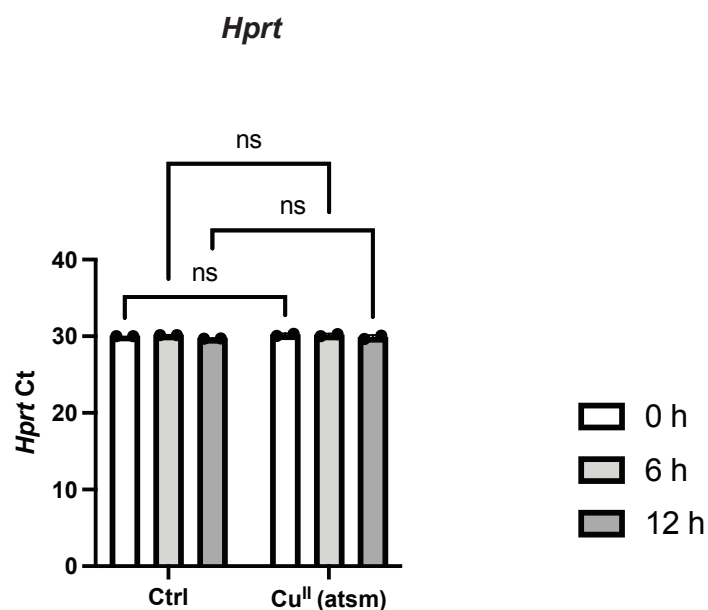

**C.**

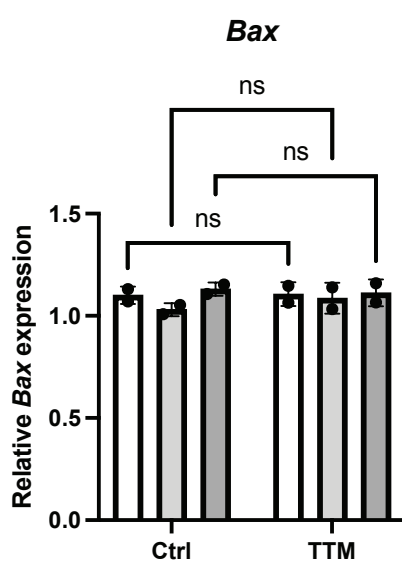

**D.**

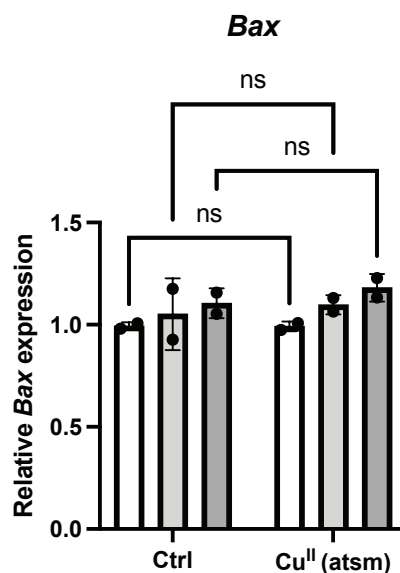

**E.**

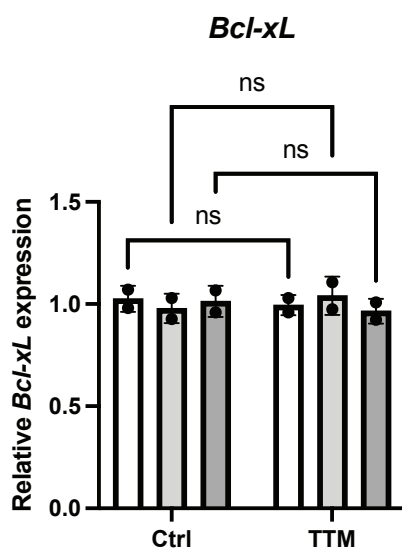

**F.**

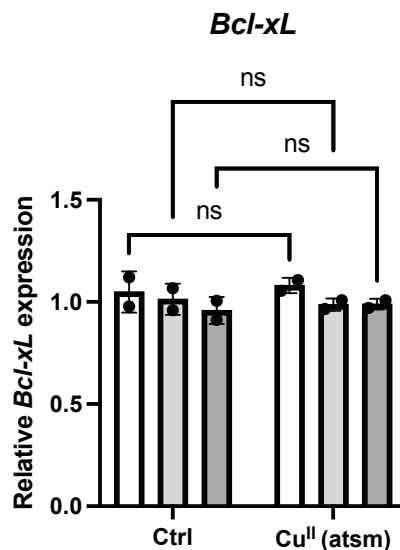

Supplement: 3 [file NIHMS2187441-supplement-3.pdf]

Supplemental Fig. 3

A.

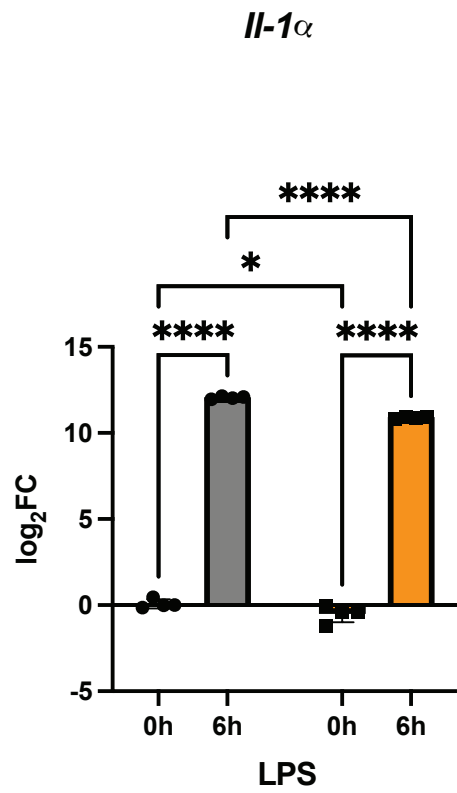

B.

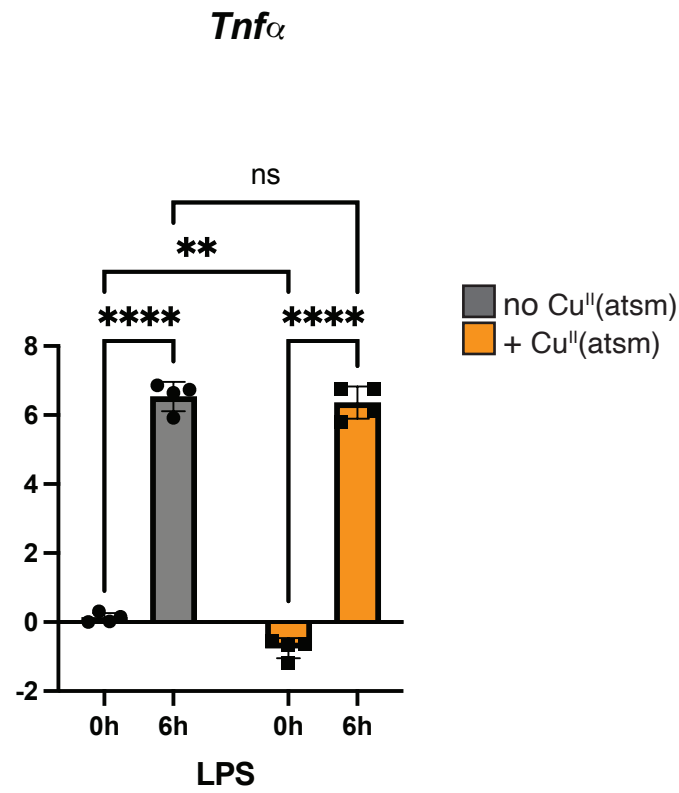

Supplement: 4 [file NIHMS2187441-supplement-4.pdf]

Supplemental Fig. 4

A.

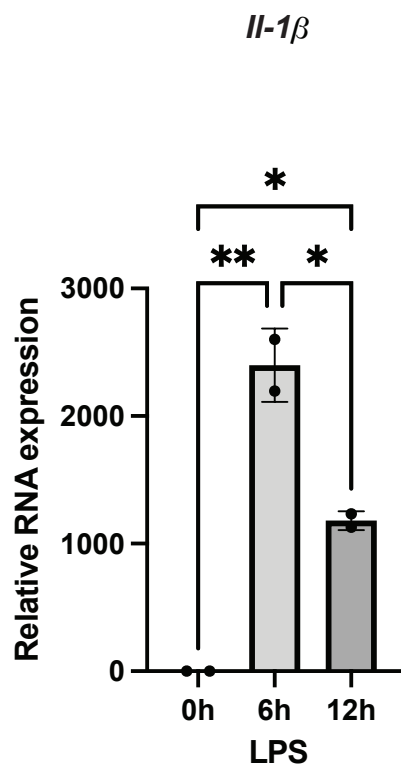

B.

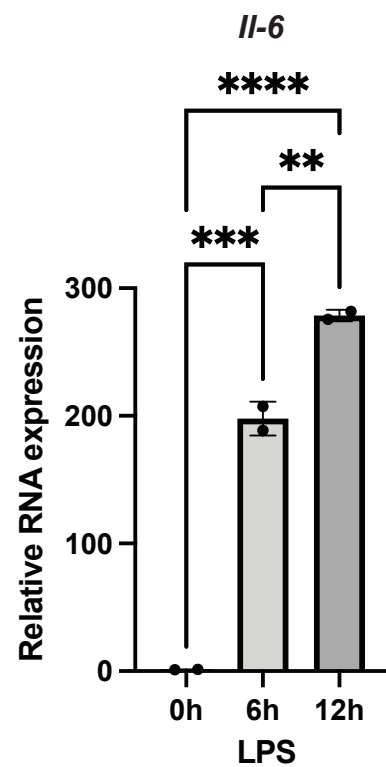

C.

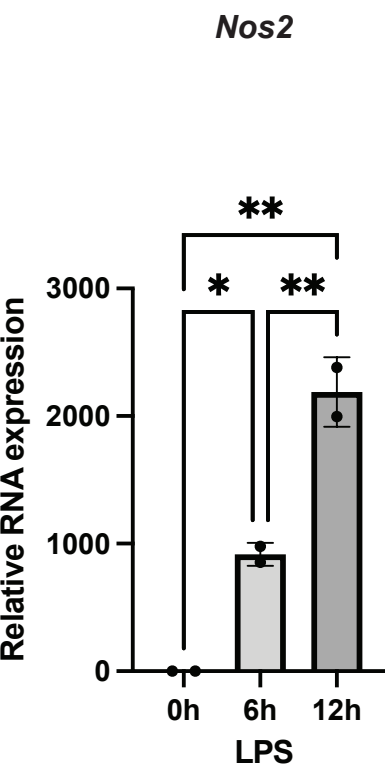

Supplement: 5 [file NIHMS2187441-supplement-5.pdf]
