# Supplementary material for: Copper regulates the expression of immune genes in microglial cells in vitro
Source: Immunobiology. Author manuscript; Available in PMC 2026 Jun 22. (PMC13285316; doi:10.1016/j.imbio.2025.153145)
Supplement: 6 [file NIHMS2187441-supplement-6.docx]

**Supplemental Information**

**Supplemental Figure Legends**

**Supplemental Fig. 1. TTM- and Cu^II^(atsm)-treated SIM-A9 cells show changes in the transcription of copper-mediated cellular stress genes**. The mRNA expressions of **A)** *Hmox1* and **B)** *Hspa1a* were determined by bulk RNA-seq and are shown as log_2_ fold changes (log_2_FC).

**Supplemental Fig. 2. TTM and Cu^II^(atsm) treatments do not induce cell death.**
SIM-A9 cells were plated at 0.5 × 10⁶ cells/well in a 12-well plate, pretreated with each compound for 1 hour, and then stimulated with LPS for 0 (white, *n*=2), 6 (light gray, *n*=2) or 12 (dark gray, *n*=2) hours. After RNA extraction and cDNA generation, the expression levels of the housekeeping gene Hprt were assessed by RT-qPCR. **A**) TTM- (F (2, 6) = 1.107, *P* = 0.3897) and **B**) Cu^II^(atsm)-treated (F (2, 6) = 0.3667, *P* = 0.7075) SIM-A9 cells exhibited comparable Hprt Ct values following compound and LPS treatment. We also examined the expressions of pro-apoptotic Bax in cells treated with **C**) TTM (F (2,6) = 0.4848, *P* = 0.6380) and **D**) Cu^II^(atsm) (F (2,6) = 0.2096, *P* = 0.8166) and anti-apoptotic Bcl-xL in cells treated with **E**) TTM (F (2, 6) = 0.7107, *P* = 0.5287) and **F**) Cu^II^(atsm) (F (2, 6) = 0.2850, *P* = 0.7617). Neither Bax nor Bcl-xL expression showed significant changes upon TTM or Cu^II^(atsm) treatment. Results are shown as as means ± standard deviation (SD). Data were analyzed by two-way ANOVA, and the results of Tukey’s multiple comparison post hoc test are indicated. ns: not significant.

**Supplemental Fig. 3. Cu^II^(atsm)-treatment induces the repression of additional proinflammatory genes.** The mRNA expressions of **A)** Il-1*α* (F (1, 12) = 3.972, *P* = 0.0695) and **B)** Tnf*α* (F (1, 12) = 3.882, *P* = 0.0723) were determined by RT-qPCR. Gray bars indicate control treatment (DMSO, *n*=4/condition), and orange bars indicate Cu^II^(atsm) (*n*=4/condition) treatment. Results are shown as mean log_2_ fold change (log_2_FC) ± standard deviation (SD). Data were analyzed using two-way ANOVA with Tukey's multiple comparison test. *p < 0.05, ***p < 0.001, and ns: not significant.

**Supplemental Fig. 4. Time course of proinflammatory gene induction by LPS in SIM-A9 cells.** The mRNA expressions of **A)** *Il-1β* (F (2, 3) = 98.60, *P* = 0.0018)*,* **B)** *Il-6* (F (2, 3) = 626.0, *P* = 0.0001), and **C)** *Nos2* (F (2, 3) = 88.11, *P* = 0.0022) were determined by RT-qPCR. White bars indicate 0 hour (*n*=2), light gray bars indicate 6 hours (*n*=2), and dark gray bars indicate 12 hours (*n*=2) of LPS treatment. Data shown is representative of three independent experiments. Results are shown as means ± SD. Data were analyzed using one-way ANOVA with Tukey's multiple comparison test. **p* < 0.05, ***p* < 0.01, ****p* < 0.001, *** *p* < 0.0001.

**Supplemental Table Legends**

**Supplemental Table 1.** Differentially expressed genes in SIM-A9 cells following treatment with LPS and Cu^II^(atsm).

**Supplemental Table 2.** Differentially expressed genes in SIM-A9 cells following treatment with LPS and TTM.

**Supplemental Table 3.** Differentially expressed genes in SIM-A9 cells following treatment with LPS and either Cu^II^(atsm) or TTM.

**Supplemental Table 4.** List of the RT-qPCR primer sequences.

**Supplemental Table 5**. List of the shRNA sequences.

**ANOVA tables**

**Fig. 1**

Fig. 1B. *Il-1β*

| ANOVA table | **SS** | **DF** | **MS** | **F (DFn, DFd)** | ***P-value*** |
| --- | --- | --- | --- | --- | --- |
| Interaction | 16.54 | 1 | 16.54 | F (1, 20) = 13.95 | *P* = 0.0013 |
| Row Factor | 530.9 | 1 | 530.9 | F (1, 20) = 447.8 | P < 0.0001 |
| Column Factor | 18.75 | 1 | 18.75 | F (1, 20) = 15.81 | *P* = 0.0007 |
| Residual | 23.71 | 20 | 1.186 |  |  |

Fig. 1C. *Il-6*

| ANOVA table | **SS** | **DF** | **MS** | **F (DFn, DFd)** | ***P-value*** |
| --- | --- | --- | --- | --- | --- |
| Interaction | 16.10 | 1 | 16.10 | F (1, 20) = 51.79 | *P* < 0.0001 |
| Row Factor | 628.6 | 1 | 628.6 | F (1, 20) = 2022 | *P* < 0.0001 |
| Column Factor | 14.55 | 1 | 14.55 | F (1, 20) = 46.81 | *P* < 0.0001 |
| Residual | 6.217 | 20 | 0.3109 |  |  |

Fig. 1D. *Nos2*

| ANOVA table | **SS** | **DF** | **MS** | **F (DFn, DFd)** | ***P-value*** |
| --- | --- | --- | --- | --- | --- |
| Interaction | 21.01 | 1 | 21.01 | F (1, 20) = 22.66 | *P* = 0.0001 |
| Row Factor | 753.1 | 1 | 753.1 | F (1, 20) = 812.1 | *P* < 0.0001 |
| Column Factor | 16.02 | 1 | 16.02 | F (1, 20) = 17.27 | *P* = 0.0005 |
| Residual | 18.55 | 20 | 0.9273 |  |  |

**Fig. 2**

Fig. 2C. *Slc31a1* (One-way ANOVA)

| ANOVA table | **SS** | **DF** | **MS** | **F (DFn, DFd)** | ***P-value*** |
| --- | --- | --- | --- | --- | --- |
| Treatment | 0.4298 | 2 | 0.2149 | F (2, 3) = 398.3 | *P* = 0.0002 |
| Residual | 0.001619 | 3 | 0.0005396 |  |  |
| Total | 0.4314 | 5 |  |  |  |

Fig. 2D. *Il-1β*

| ANOVA table | **SS** | **DF** | **MS** | **F (DFn, DFd)** | ***P-value*** |
| --- | --- | --- | --- | --- | --- |
| Interaction | 1.278 | 2 | 0.6390 | F (2, 18) = 3.551 | *P* = 0.0501 |
| Row Factor | 760.4 | 1 | 760.4 | F (1, 18) = 4226 | *P* < 0.0001 |
| Column Factor | 3.356 | 2 | 1.678 | F (2, 18) = 9.323 | *P* = 0.0017 |
| Residual | 3.239 | 18 | 0.1800 |  |  |

Fig. 2E. *Il-6*

| ANOVA table | **SS** | **DF** | **MS** | **F (DFn, DFd)** | ***P-value*** |
| --- | --- | --- | --- | --- | --- |
| Interaction | 3.611 | 2 | 1.805 | F (2, 18) = 13.50 | *P* = 0.0003 |
| Row Factor | 230.8 | 1 | 230.8 | F (1, 18) = 1726 | *P* < 0.0001 |
| Column Factor | 5.135 | 2 | 2.567 | F (2, 18) = 19.20 | *P* < 0.0001 |
| Residual | 2.407 | 18 | 0.1337 |  |  |

Fig. 2F. *Nos2*

| ANOVA table | **SS** | **DF** | **MS** | **F (DFn, DFd)** | ***P-value*** |
| --- | --- | --- | --- | --- | --- |
| Interaction | 17.10 | 2 | 8.551 | F (2, 18) = 66.42 | *P* < 0.0001 |
| Row Factor | 625.8 | 1 | 625.8 | F (1, 18) = 4861 | *P* < 0.0001 |
| Column Factor | 24.78 | 2 | 12.39 | F (2, 18) = 96.26 | *P* < 0.0001 |
| Residual | 2.317 | 18 | 0.1287 |  |  |

**Fig. 3**

Fig. 3B. *Il-1β*

| ANOVA table | **SS** | **DF** | **MS** | **F (DFn, DFd)** | ***P-value*** |
| --- | --- | --- | --- | --- | --- |
| Interaction | 5.574 | 1 | 5.574 | F (1, 20) = 7.187 | *P* = 0.0144 |
| Row Factor | 696.6 | 1 | 696.6 | F (1, 20) = 898.1 | *P* < 0.0001 |
| Column Factor | 27.74 | 1 | 27.74 | F (1, 20) = 35.77 | *P* < 0.0001 |
| Residual | 15.51 | 20 | 0.7756 |  |  |

Fig. 3C. *Il-6*

| ANOVA table | **SS** | **DF** | **MS** | **F (DFn, DFd)** | ***P-value*** |
| --- | --- | --- | --- | --- | --- |
| Interaction | 27.62 | 1 | 27.62 | F (1, 20) = 31.99 | *P* < 0.0001 |
| Row Factor | 209.9 | 1 | 209.9 | F (1, 20) = 243.1 | *P* < 0.0001 |
| Column Factor | 40.89 | 1 | 40.89 | F (1, 20) = 41.35 | *P* < 0.0001 |
| Residual | 17.27 | 20 | 0.8636 |  |  |

Fig.3D. *Nos2*

| ANOVA table | **SS** | **DF** | **MS** | **F (DFn, DFd)** | ***P-value*** |
| --- | --- | --- | --- | --- | --- |
| Interaction | 26.59 | 1 | 26.59 | F (1, 20) = 8.207 | *P* = 0.0098 |
| Row Factor | 304.7 | 1 | 304.7 | F (1, 20) = 94.04 | *P* < 0.0001 |
| Column Factor | 61.76 | 1 | 61.76 | F (1, 20) = 19.06 | *P* = 0.0003 |
| Residual | 64.81 | 20 | 3.241 |  |  |

Fig.3E. *Atp7a* (one-way ANOVA)

| ANOVA table | **SS** | **DF** | **MS** | **F (DFn, DFd)** | ***P-value*** |
| --- | --- | --- | --- | --- | --- |
| Treatment | 0.07146 | 2 | 0.03573 | F (2, 3) = 75.46 | *P* = 0.0027 |
| Residual | 0.001421 | 3 | 0.0004735 |  |  |
| Total | 0.07288 | 5 |  |  |  |

**Supplemental Fig. 2**

Supplemental Fig. 2A. *Hprt* (TTM-treated cells)

| ANOVA table | **SS** | **DF** | **MS** | **F (DFn, DFd)** | ***P-value*** |
| --- | --- | --- | --- | --- | --- |
| Interaction | 0.1797 | 2 | 0.08985 | F (2, 6) = 1.107 | *P* = 0.3897 |
| Row Factor | 0.07553 | 1 | 0.07553 | F (1, 6) = 0.9306 | *P* = 0.3720 |
| Column Factor | 0.4520 | 2 | 0.2260 | F (2, 6) = 2.785 | *P* = 0.1395 |
| Residual | 0.4869 | 6 | 0.08116 |  |  |

Supplemental Fig. 2B. *Hprt* (Cu^II^(atsm)-treated cells)

| ANOVA table | **SS** | **DF** | **MS** | **F (DFn, DFd)** | ***P-value*** |
| --- | --- | --- | --- | --- | --- |
| Interaction | 0.01989 | 2 | 0.009944 | F (2, 6) = 0.3667 | *P* = 0.7075 |
| Row Factor | 0.03564 | 1 | 0.03564 | F (1, 6) = 1.315 | *P* = 0.2952 |
| Column Factor | 0.3108 | 2 | 0.1554 | F (2, 6) = 5.731 | *P* = 0.0406 |
| Residual | 0.1627 | 6 | 0.02711 |  |  |

Supplemental Fig. 2C. *Bax* (TTM-treated cells)

| ANOVA table | **SS** | **DF** | **MS** | **F (DFn, DFd)** | ***P-value*** |
| --- | --- | --- | --- | --- | --- |
| Interaction | 0.002800 | 3 | 0.001400 | F (2, 6) = 0.4848 | *P* = 0.6380 |
| Row Factor | 0.0006253 | 1 | 0.0006253 | F (1, 6) = 1.315 | *P* = 0.2952 |
| Column Factor | 0.008403 | 2 | 0.004201 | F (2, 6) = 1.455 | *P* = 0.3053 |
| Residual | 0.01732 | 6 | 0.002887 |  |  |

Supplemental Fig. 2D. *Bax* (Cu^II^(atsm)-treated cells)

| ANOVA table | **SS** | **DF** | **MS** | **F (DFn, DFd)** | ***P-value*** |
| --- | --- | --- | --- | --- | --- |
| Interaction | 0.003062 | 2 | 0.001531 | F (2, 6) = 0.2096 | *P* = 0.8166 |
| Row Factor | 0.004757 | 1 | 0.004757 | F (1, 6) = 0.6515 | *P* = 0.4505 |
| Column Factor | 0.04531 | 2 | 0.02265 | F (2, 6) = 3.102 | *P* = 0.1188 |
| Residual | 0.04382 | 6 | 0.007303 |  |  |

Supplemental Fig. 2E. *Bcl-xL* (Cu^II^(atsm)-treated cells)

| ANOVA table | **SS** | **DF** | **MS** | **F (DFn, DFd)** | ***P-value*** |
| --- | --- | --- | --- | --- | --- |
| Interaction | 0.007069 | 2 | 0.003534 | F (2, 6) = 0.7101 | *P* = 0.5287 |
| Row Factor | 9.053e-005 | 1 | 9.053e-005 | F (1, 6) = 0.01819 | *P* = 0.8971 |
| Column Factor | 0.001124 | 2 | 0.0005619 | F (2, 6) = 0.1129 | *8951* |
| Residual | 0.02986 | 6 | 0.004977 |  |  |

Supplemental Fig. 2F. *Bcl-xL*(Cu^II^(atsm)-treated cells)

| ANOVA table | **SS** | **DF** | **MS** | **F (DFn, DFd)** | ***P-value*** |
| --- | --- | --- | --- | --- | --- |
| Interaction | 0.002211 | 2 | 0.001105 | F (2, 6) = 0.2850 | *P* = 0.7617 |
| Row Factor | 0.0004229 | 1 | 0.0004229 | F (1, 6) = 0.1090 | *P* = 0.7525 |
| Column Factor | 0.01753 | 2 | 0.008767 | F (2, 6) = 2.260 | *P* = 0.1855 |
| Residual | 0.02327 | 6 | 0.003879 |  |  |

**Supplemental Fig. 3**

Supplemental Fig. 3A. *Il-1α*

| ANOVA table | **SS** | **DF** | **MS** | **F (DFn, DFd)** | ***P-value*** |
| --- | --- | --- | --- | --- | --- |
| Interaction | 0.3159 | 1 | 0.3159 | F (1, 12) = 3.972 | *P* = 0.0695 |
| Row Factor | 547.1 | 1 | 547.1 | F (1, 12) = 6879 | *P* < 0.0001 |
| Column Factor | 3.048 | 1 | 3.048 | F (1, 12) = 38.33 | *P* < 0.0001 |
| Residual | 0.9544 | 12 | 0.07953 |  |  |

Supplemental Fig. 3B. *Tnfα*

| ANOVA table | **SS** | **DF** | **MS** | **F (DFn, DFd)** | ***P-value*** |
| --- | --- | --- | --- | --- | --- |
| Interaction | 0.4881 | 1 | 0.4881 | F (1, 12) = 3.882 | *P* = 0.0723 |
| Row Factor | 183.2 | 1 | 183.2 | F (1, 12) = 1457 | *P* < 0.0001 |
| Column Factor | 1.115 | 1 | 1.115 | F (1, 12) = 8.864 | *P* = 0.0115 |
| Residual | 1.509 | 12 | 0.1257 |  |  |

**Supplemental Fig. 4**

Supplemental Fig. 4A. *Il-1β*  (One-way ANOVA)

| ANOVA table | **SS** | **DF** | **MS** | **F (DFn, DFd)** | ***P-value*** |
| --- | --- | --- | --- | --- | --- |
| Treatment | 5749172 | 2 | 2874586 | F (2, 3) = 98.60 | *P* = 0.0018 |
| Residual | 87462 | 3 | 29154 |  |  |
| Total | 5836634 | 4 |  |  |  |

Supplemental Fig. 4B. *Il-6* (One-way ANOVA)

| ANOVA table | **SS** | **DF** | **MS** | **F (DFn, DFd)** | ***P-value*** |
| --- | --- | --- | --- | --- | --- |
| Treatment | 81490 | 2 | 40745 | F (2, 3) = 626.0 | *P* = 0.0001 |
| Residual | 195.3 | 3 | 65.09 |  |  |
| Total | 81686 | 5 |  |  |  |

Supplemental Fig. 4C. *Nos2* (One-way ANOVA)

| ANOVA table | **SS** | **DF** | **MS** | **F (DFn, DFd)** | ***P-value*** |
| --- | --- | --- | --- | --- | --- |
| Treatment | 4829336 | 2 | 2414668 | F (2, 3) = 88.11 | *P* = 0.0022 |
| Residual | 82212 | 3 | 27404 |  |  |
| Total | 4911548 | 5 |  |  |  |

SS: Sum of Squares, DF: Degrees of Freedom, MS: Mean Square,

F: F-ratio, DFn: numerator DF, DFd: denominator DF
